# Epigenetic silencing of *miR-340-5p* in multiple myeloma: mechanisms and prognostic impact

**DOI:** 10.1186/s13148-019-0669-2

**Published:** 2019-05-07

**Authors:** Zhenhai Li, Kwan Yeung Wong, George A. Calin, Wee-Joo Chng, Godfrey Chi-fung Chan, Chor Sang Chim

**Affiliations:** 1 Department of Medicine, Queen Mary Hospital, The University of Hong Kong, Pokfulam Road, Pokfulam, Hong Kong; 2 Department of Experimental Therapeutics, The University of Texas MD Anderson Cancer Center, Houston, TX USA; 3 Cancer Science Institute of Singapore, National University of Singapore, Singapore, Singapore; 4 Department of Haematology-Oncology, National University Cancer Institute, Singapore, Singapore; 5 Department of Medicine, Yong Loo Lin School of Medicine, National University of Singapore, Singapore, Singapore; 6 Department of Pediatrics and Adolescent Medicine, Queen Mary Hospital, The University of Hong Kong, Pokfulam, Hong Kong

**Keywords:** Multiple myeloma, miR-340-5p, DNA methylation, XIAP, Overall survival

## Abstract

**Background:**

*miR-340-5p*, localized to 5q35, is a tumor suppressor miRNA implicated in multiple cancers. As a CpG island is present at the putative promoter region of its host gene, *RNF130*, we hypothesized that the intronic *miR-340-5p* is a tumor suppressor miRNA epigenetically silenced by promoter DNA methylation of its host gene in multiple myeloma.

**Results:**

By pyrosequencing-confirmed methylation-specific PCR, *RNF130/miR-340* was methylated in 8/15 (53.3%) myeloma cell lines but not normal plasma cells. Methylation correlated inversely with the expression of both *miR-340-5p* and *RNF130*. In completely methylated WL-2 and RPMI-8226R cells, 5-AzadC treatment led to demethylation and re-expression of *miR-340-5p*. In primary samples, *RNF130/miR-340* methylation was detected in 4 (22.2%) monoclonal gammopathy of undetermined significance, 15 (23.8%) diagnostic myeloma, and 7 (23.3%) relapsed myeloma. *RNF130/miR-340* methylation at diagnosis was associated with inferior overall survival (median 27 vs. 68 months; *P =* 3.944E−5). In WL-2 cells, overexpression of *miR-340-5p* resulted in reduced cellular proliferation [MTS, *P* = 0.002; verified in KMS-12-PE (*P* = 0.002) and RPMI-8226R (*P* = 2.623E−05) cells], increased cell death (trypan blue, *P* = 0.005), and enhanced apoptosis by annexin V-PI staining. Moreover, by qRT-PCR, overexpression of *miR-340-5p* led to repression of both known targets (*CCND1* and *NRAS*) and bioinformatically predicted targets in MAPK signaling (*MEKK1*, *MEKK2*, and *MEKKK3*) and apoptosis (*MDM4* and *XIAP*), hence downregulation of phospho-ERK1/2 and XIAP by Western blot. Furthermore, by qRT-PCR, in CD138-sorted primary samples (*n* = 37), *miR-340-5p* and *XIAP* were inversely correlated (*P* = 0.002). By luciferase assay, XIAP was confirmed as a direct target of *miR-340-5p* via targeting of the distal but not proximal seed region binding site.

**Conclusions:**

Collectively, tumor-specific methylation-mediated silencing of *miR-340-5p* is likely an early event in myelomagenesis with adverse survival impact, via targeting multiple oncogenes in MAPK signaling and apoptosis, thereby a tumor suppressive miRNA in myeloma.

**Electronic supplementary material:**

The online version of this article (10.1186/s13148-019-0669-2) contains supplementary material, which is available to authorized users.

## Background

Multiple myeloma is the second most common blood cancer, accounting for approximately 10% of all hematologic malignancies [1]. Patients with myeloma are often preceded by an asymptomatic pre-malignant stage of monoclonal gammopathy of undetermined significance (MGUS), which progresses into symptomatic myeloma at a rate of 1% per year [2]. Genetically, about half of patients with myeloma carries a non-hyperdiploid karyotype, which is associated with recurrent translocations involving immunoglobulin gene located at 14q32, whereas the other half carries a hyperdiploid (HPRD) karyotype, characterized by trisomies of odd number chromosomes [3]. Apart from chromosomal copy number alterations, recurrent genetic mutations have been identified, in particular, amongst genes involved in RAS (*NRAS*, *KRAS*, and *BRAF*), NF-kB (*TRAF3*, *CYLD*, and *LTB*), and TP53 (*TP53*, *ATM*, and *ATR*) signaling pathways [4–6].

MicroRNAs (miRNAs) are endogenous, single-stranded, small non-coding RNAs of ~ 22 nt in length [7, 8]. Functionally, a miRNA will target and suppress the expression of their target protein-coding genes by complementary binding of the seed region, i.e., the second to seventh nucleotides, to the seed region binding site (SRBS) in the 3′-UTR of the targeted mRNAs of protein-coding genes, leading to translational blockade or mRNA degradation [9]. Deregulated miRNA expression has been found in cancers including hematologic malignancies [10, 11], in which oncogenic miRNAs targeting tumor suppressor genes are upregulated, whereas tumor suppressive miRNAs targeting oncogenes are downregulated [8, 11]. For instance, overexpression of *miR-21*, of which PTEN was a direct target, led to the activation of PI3K/AKT signaling pathway, and hence, *miR-21* is an oncomiR promoting cellular proliferation [12]. On the other hand, *miR-30c*, targeting BCL9, has been shown downregulated in myeloma. BCL9 is an essential effector component for transcription of oncogenic Wnt target genes [13]. Moreover, restoration of *miR-30c* led to inhibition of cell proliferation, invasion, and migration in addition to enhancing apoptosis of myeloma cells, hence a tumor suppressor miRNA [14].

DNA methylation refers to the addition of a methyl (-CH_3_) group to carbon five position of the cytosine ring in a CpG dinucleotide [15]. CpG dinucleotides may cluster as a CpG island, which is defined as any genomic region of > 200 bp with a high GC content of > 50% and a high ratio of observed/expected CpG > 0.60 [16, 17]. In the mammalian genome, promoter-associated CpG islands are localized to or in close proximity to the promoter region of more than half of the human genes [18] and involved in the regulation of gene expression by DNA methylation [19]. In normal cells, the majority of promoter-associated CpG islands are unmethylated, associated with a euchromatin configuration, and hence transcriptionally ready or active for gene expression [20]. Conversely, CpG islands/sites in the intergenic regions, imprinted regions, and X-chromosome are hypermethylated, leading to repression of repetitive elements, such as SINE and LINE elements, imprinted genes, and X-linked genes respectively [19]. In contrast to normal cells, cancer cells are characterized by global DNA hypomethylation and locus-specific hypermethylation of promoter-associated CpG islands of tumor suppressor genes or miRNAs, resulting in downregulation, and hence loss of tumor suppressor functions [8, 21, 22]. For instance, in myeloma, tumor suppressive *miR-34b/c* [23], *miR-203* [24], and *miR-129-2* [25] have been shown to be silenced by promoter DNA methylation. Moreover, epigenetic silencing of *miR-137* has been found to correlate with shorter progression-free survival in myeloma [26].

*miR-340-5p*, localized to 5q35, is embedded in the second intron of its host gene, *RNF130*, and has been shown to be a tumor suppressor downregulated in several cancers, such as breast cancer [27], ovarian cancer [28], and hepatocellular carcinoma [29]. In hepatocellular carcinoma, overexpression of *miR-340-5p* by oligo transfection resulted in inhibition of cell proliferation, migration, and invasion in vitro and suppression of tumor growth in vivo by directly targeting Janus kinase 1 [29]. As a CpG island is present at the promoter region of its host gene, *RNF130*, we hypothesized that *miR-340-5p* is an intronic tumor suppressor miRNA epigenetically silenced by *RNF130/miR-340* promoter DNA hypermethylation in multiple myeloma (Additional file 3: Figure S3).

## Results

### Methylation of *RNF130/miR-340* in healthy controls and human myeloma cell lines (HMCLs)

**Fig. 1:**
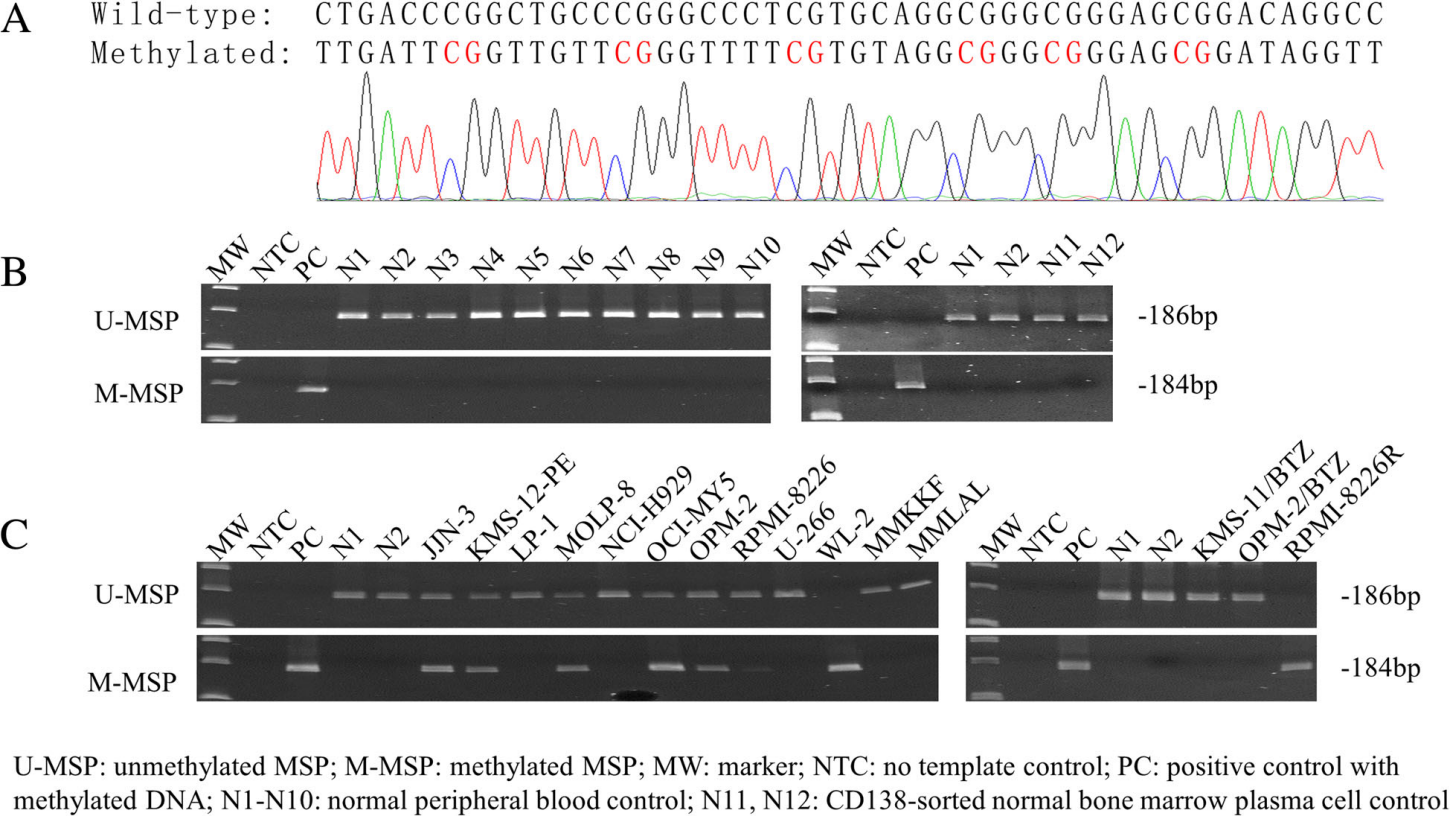
Methylation of *RNF130/miR-340* in healthy controls and HMCLs. **a** Direct sequencing of M-MSP products from positive control with methylated DNA showed the conversion of all unmethylated cytosines into uracils (turned into thymidines after PCR) but all methylated cytosines remained unchanged, indicating the completeness of bisulfite conversion and specificity of MSP. **b** M-MSP and U-MSP showed all healthy controls (N1-N12) were completely unmethylated (UU), whereas positive control with methylated DNA was completely methylated (MM). **c** M-MSP and U-MSP showed *RNF130/miR-340* was MM in HMCLs, including WL-2 and RPMI-8226R; partially methylated (MU) in JJN-3, KMS-12-PE, MOLP-8, OCI-MY5, OPM-2, and RPMI-8226; and UU in LP-1, NCI-H929, U-266, MMKKF, MMLAL, KMS-11/BTZ, and OPM-2/BTZ

Methylation-specific PCR (MSP) was carried out to examine methylation of *RNF130/miR-340* in the bisulfite-converted DNA of healthy controls [peripheral blood (*n* = 10) and CD138-sorted bone marrow plasma cell (*n* = 2)] and HMCLs (*n* = 15). Direct sequencing of the M-MSP products from positive control with methylated DNA confirmed the completeness of bisulfite conversion and MSP specificity, as indicated by conversion of all unmethylated cytosines into thymidines after PCR, whereas all methylated cytosines remained unchanged (Fig. 1a). None of the healthy controls showed methylation of *RNF130/miR-340* (Fig. 1b). By contrast, in HMCLs, *RNF130/miR-340* was completely methylated (MM; M-MSP positive but U-MSP negative) in WL-2 and RPMI-8226R; partially methylated (MU; both M-MSP and U-MSP positive) in JJN-3, KMS-12-PE, MOLP-8, OCI-MY5, OPM-2, and RPMI-8226; and completely unmethylated (UU; M-MSP negative but U-MSP positive) in LP-1, NCI-H929, U-266, MMKKF, MMLAL, KMS-11/BTZ, and OPM-2/BTZ (Fig. 1c). Moreover, these MSP methylation statuses (MM, MU, and UU) were verified using quantitative bisulfite pyrosequencing, which showed that completely methylated HMCLs were associated with a higher methylation level between 62.8 and 94.6%, partially methylated HMCLs carried an intermediate methylation level of 17.0 to 35.4%, and completely unmethylated HMCLs were associated with a lower methylation level from 5.2 to 8.6% (Additional file 1: Figure S1). These data suggested that *RNF130/miR-340* was methylated in a tumor-specific manner in myeloma.

### Methylation and expression of *miR-340-5p* and its host gene *RNF130* in HMCLs

**Fig. 2:**
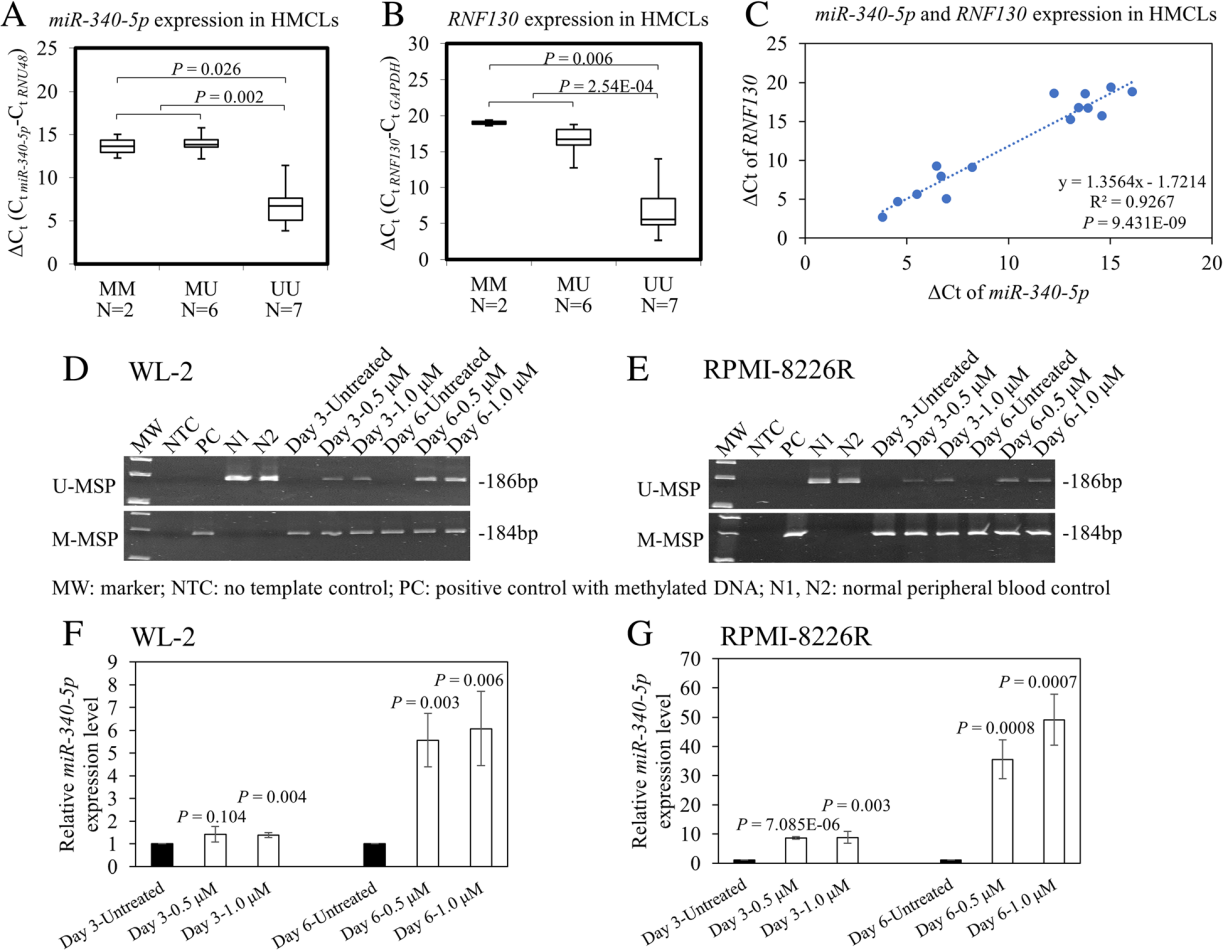
Methylation and expression of *miR-340-5p* and its host gene, *RNF130*, in HMCLs. By qRT-PCR, methylation of *RNF130/miR-340* was significantly correlated with lower expression level and hence larger ΔCt of both **a** *miR-340-5p* (MM vs. UU, *P* = 0.026; MM + MU vs. UU, *P* = 0.002) and **b** its host gene, *RNF130* (MM vs. UU, *P* = 0.006; MM + MU vs. UU, *P* = 2.54E−4). **c** In HMCLs, using ΔCt, the expression of *RNF130* was plotted against *miR-340-5p*, and hence a concordant expression of *miR-340-5p* and its host gene was shown. In WL-2 cells (**d**) and RPMI-8226R cells (**e**), both completely methylated for *RNF130/miR-340*, treatment with 5-AzadC led to *RNF130/miR-340* promoter demethylation, as evidenced by the emergence of U-MSP signal and concomitant re-expression of mature *miR-340-5p* in WL-2 cells (**f**) and RMPI-8226R cells (**g**). Error bars represent the standard deviation from three independent qRT-PCR

To study if methylation of *RNF130/miR-340* was correlated with downregulated expression of *miR-340-5p* and its host gene, *RNF130*, qRT-PCR was employed to measure the expression levels of *miR-340-5p* and *RNF130* in HMCLs. Results showed that HMCLs with methylation of *RNF130/miR-340* had significantly lower expression levels of both *miR-340-5p* (Fig. 2a; MM vs. UU, *P* = 0.026; MM + MU vs. UU, *P* = 0.002) and *RNF130* (Fig. 2b; MM vs. UU, *P* = 0.006; MM + MU vs. UU, *P* = 2.54E−4) than HMCLs completely unmethylated for *RNF130/miR-340*. Moreover, to examine if *miR-340-5p* was co-expressed with *RNF130*, in HMCLs, the expression of *miR-340-5p* was plotted against *RNF130*, and a concordant expression of *miR-340-5p* and *RNF130* was demonstrated (Fig. 2c; *R*^2^ = 0.9267, *P* = 9.431E−09).

To further testify if promoter DNA methylation resulted in downregulation of *miR-340-5p*, WL-2 and RPMI-8226R cells, which were completely methylated for *RNF130/miR-340*, were treated with 5-AzadC, a demethylation agent. Upon treatment with 5-AzadC, the promoter of *RNF130/miR-340* was demethylated as evidenced by the emergence of U-MSP signal on both day 3 and day 6 (Fig. 2d, e). Moreover, by qRT-PCR, mature *miR-340-5p* was simultaneously re-expressed by 1.4- to 8.8-fold on day 3 and by 5.6- to 49.1-fold on day 6 (Fig. 2f, g). Hence, in myeloma cells, methylation-mediated silencing of *miR-340-5p* was reversible.

### Methylation and expression of *RNF130/miR-340* in primary bone marrow samples of MGUS, diagnostic myeloma, and relapsed myeloma

**Fig. 3:**
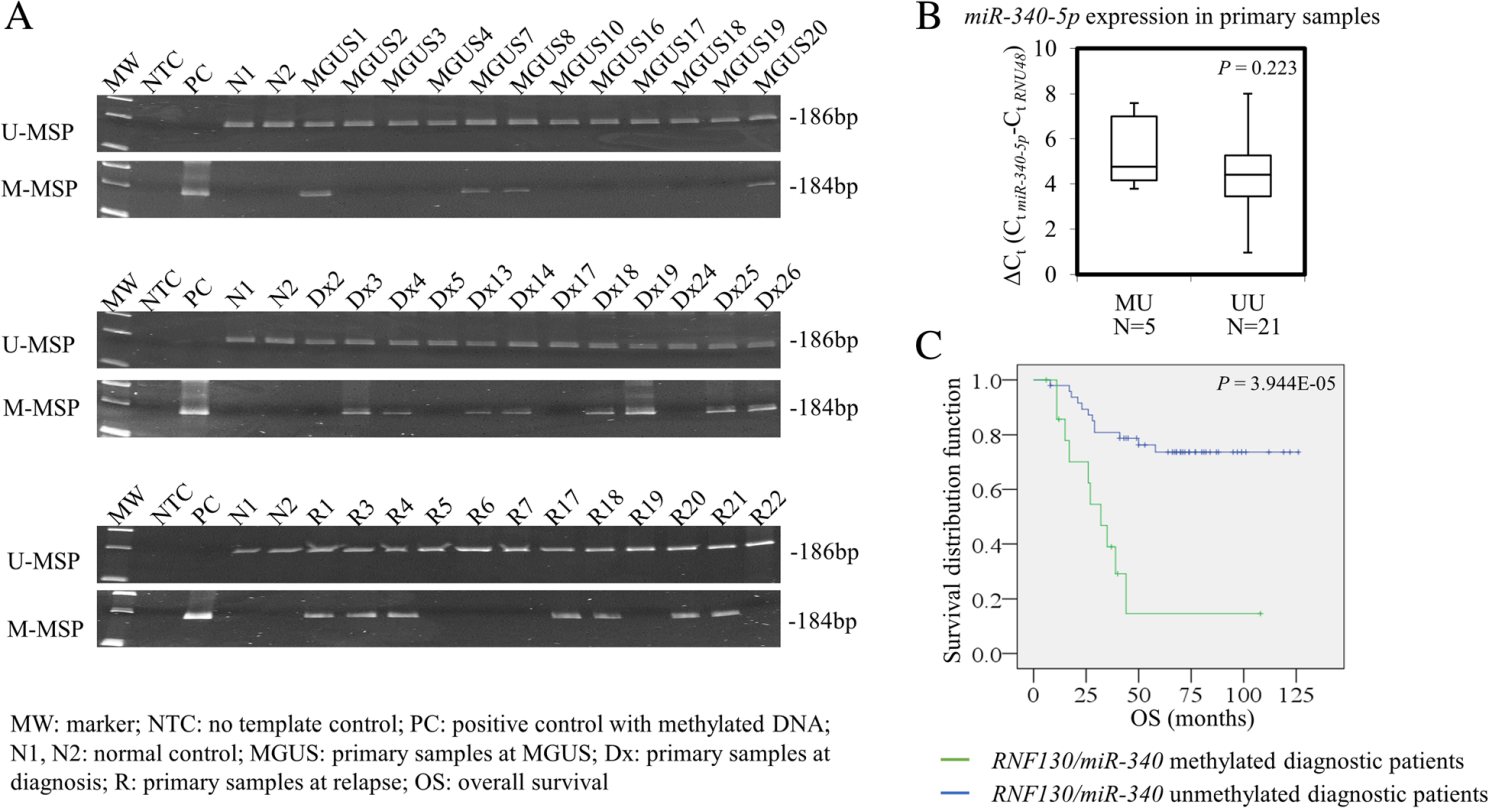
Methylation and expression of *RNF130/miR-340* in primary bone marrow samples. **a** Representative M-MSP and U-MSP showing methylation of *RNF130/miR-340-5p* in primary samples of MGUS, diagnostic myeloma, and relapsed myeloma. The numbers were assigned for illustration purpose, and hence, the identical Arabic numerals in different disease stages are not serial samples from the same patient. **b** In 26 patients with both CD138-sorted DNA and RNA, there was a trend that patients with methylation of *RNF130/miR-340* (*n* = 5) had a lower expression and hence larger ΔCt of mature *miR-340-5p* than patients without methylation (*n* = 21). **c** Kaplan-Meier analysis of OS in patients with and without methylation of *RNF130/miR-340*

By MSP, methylation of *RNF130/miR-340* was detected in 4 (22.2%) MGUS, 15 (23.8%) diagnostic myeloma, and 7 (23.3%) relapsed myeloma primary bone marrow samples (Fig. 3a). Methylation frequency of *RNF130/miR-340* was not significantly different among those consecutive clinical stages of myeloma (MGUS vs. diagnostic myeloma: *P* = 1.000; diagnostic myeloma vs. relapsed myeloma: *P* = 1.000). However, in contrast to the absence of methylation in normal, the appearance of methylation in MGUS and a comparable frequency in consecutive stages from MGUS to diagnostic myeloma and to relapsed myeloma indicated it might be an early event in the pathogenesis of myeloma. Moreover, among 26 diagnostic samples with paired CD138-sorted RNA samples, methylation of *RNF130/miR-340* had a trend of associating with lower expression of *miR-340-5p* (Fig. 3b; *P* = 0.223).

Interestingly, by Kaplan-Meier analysis, the projected overall survival (OS) of diagnostic myeloma patients with and without *RNF130/miR-340* methylation was 33.3% and 75.0% respectively, and patients with *RNF130/miR-340* methylation (*n* = 15) showed significantly shorter OS than patients without *RNF130/miR-340* methylation (*n* = 48) (Fig. 3c; median 27 vs. 68 months; *P* = 3.944E−5). By Cox regression multivariate analysis, *miR-340-5p* methylation (*P* = 0.002; hazard ratio 8.983; 95% confidence interval 2.203–36.630) was shown to be an adverse prognostic factor of OS independent of ISS (*P* = 0.652), age (*P* = 0.331), sex (*P* = 0.690), isotype (*P* = 0.198), high LDH (*P* = 0.367), and high-risk karyotype (*P* = 0.438) in primary myeloma samples at diagnosis. Moreover, methylation of *RNF130/miR-340* was preferentially detected in IgD and IgG myeloma (*P* = 0.016), but not associated with gender (*P* = 0.383), age (*P* = 0.933), or ISS stage (*P* = 0.985).

### Tumor suppressive function of *miR-340-5p* in myeloma cells

**Fig. 4:**
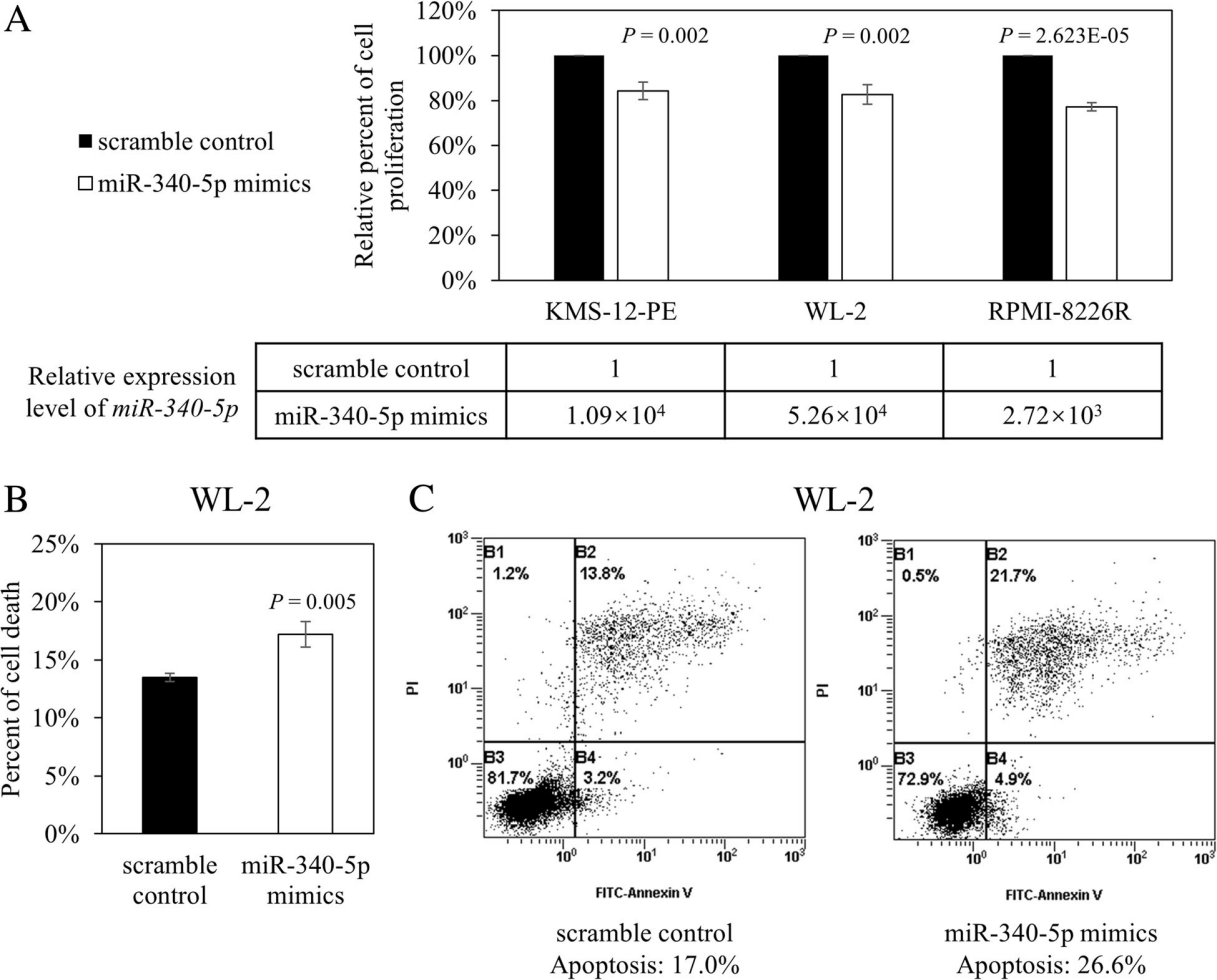
Restoration of *miR-340-5p* in HMCLs. **a** By qRT-PCR, *miR-340-5p* was shown to be successfully overexpressed in KMS-12-PE, WL-2, and RPMI-8226R cells. By MTS assay, overexpression of *miR-340-5p* significantly inhibited cellular proliferation in all three cell lines. **b** By trypan blue staining, overexpression of *miR-340-5p* increased the number of dead cells by 3.73% in WL-2 cells. Error bars represent the standard deviation from three independent transfections. **c** By annexin V/PI analysis, overexpression of *miR-340-5p* increased cellular apoptosis by 9.60% in WL-2 cells

As methylation-mediated silencing of *miR-340-5p* was frequently detected in HMCLs and primary samples, we postulated that it might act as a tumor suppressor. By MTS assay, as compared to scramble control, overexpression of *miR-340-5p* resulted in reduced cellular proliferation in KMS-12-PE by 15.7% (*P* = 0.002), WL-2 by 17.3% (*P* = 0.002), and RPMI-8226R by 22.8% (*P* = 2.623E−05) (Fig. 4a). Moreover, in WL-2 cells, an increase of dead cells by 3.73% (Fig. 4b; *P* = 0.005) and induction of apoptosis by 9.60% (Fig. 4c) were also demonstrated using trypan blue staining and annexin V-PI analysis respectively, suggesting a tumor suppressive role of *miR-340-5p* in myeloma cells.

### Overexpression of *miR-340-5p* downregulated multiple target genes involved in MAPK signaling and apoptosis

To further elucidate the mechanism of *miR-340-5p* in the regulation of cellular proliferation and apoptosis, the expression of previously validated known target genes, including *NRAS* [30], *MDM2* [31], *NFKB1* [28], and *CCND1* [32], were studied. Moreover, by miRWalk2.0 [33], which is a bioinformatics platform combining several well-known miRNA target prediction algorithms, including miRanda, miRDB, TargetScan, and PITA, additional potential direct targets with known oncogenic properties, including *MEKK1*, *MEKK2*, *MEKKK3*, *MDM4*, and *XIAP*, were predicted and further studied.

**Fig. 5:**
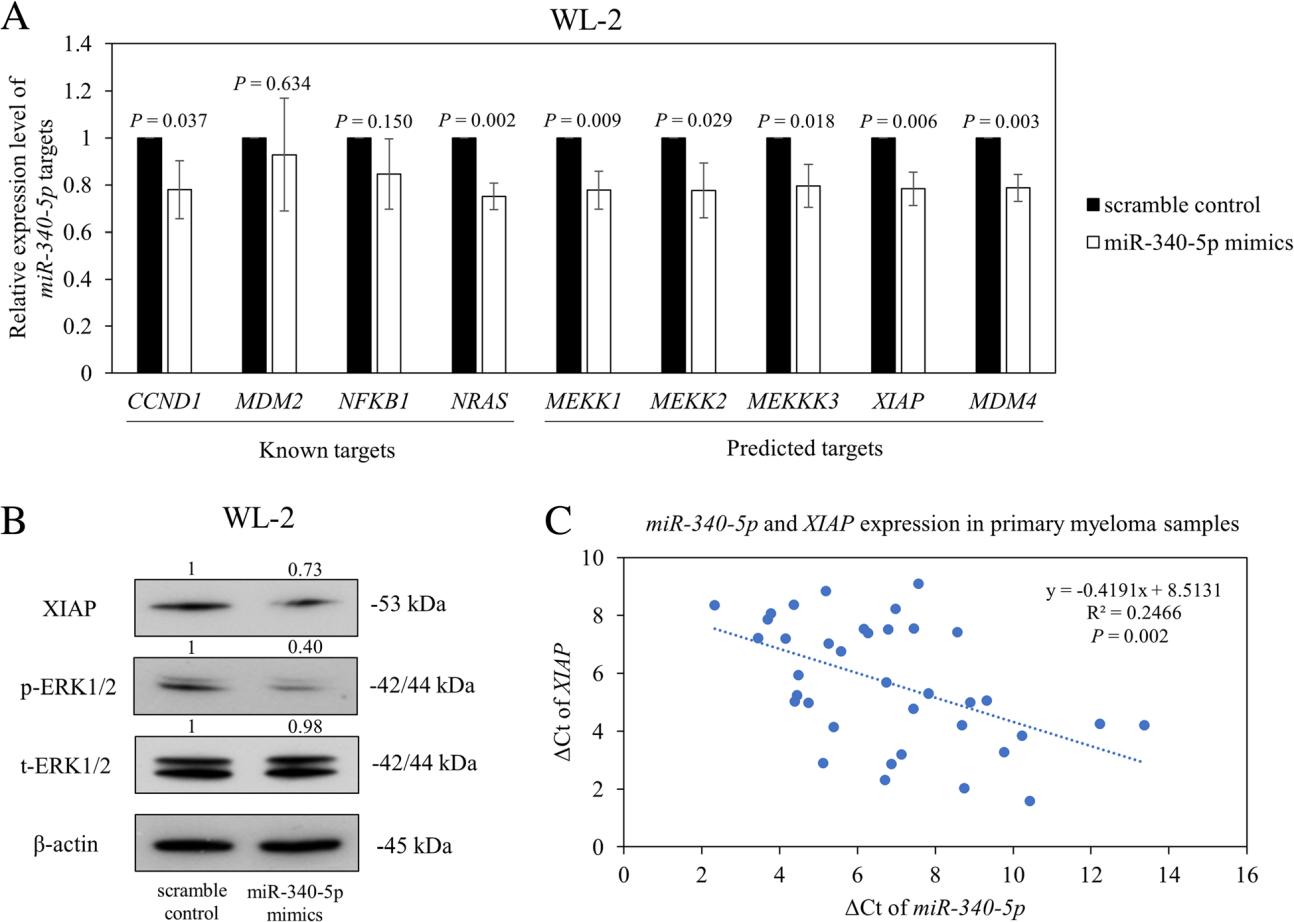
Effect of *miR-340-5p* in the regulation of target genes and signaling pathways. **a** Upon overexpression of *miR-340-5p*, relative expression levels of myeloma-related known target genes of *miR-340-5p*, including *CCND1*, *MDM2*, *NFKB1*, and *NRAS*, and bioinformatically predicted oncogenic target genes involved in MAPK (*MEKK1*, *MEKK2*, and *MEKKK3*), apoptosis (*XIAP*), and TP53 (*MDM4*) signaling pathways were shown. Error bars represent the standard deviation from three independent qRT-PCR. **b** Western blot analysis of phospho-ERK1/2, total-ERK1/2 (downstream to MAPK signaling), and XIAP upon transfection with *miR-340-5p* mimics or scramble control were shown. β-Actin was set as the endogenous control and normalizer for densitometric analysis of protein levels. Relative normalized protein levels are shown above the corresponding band. **c** In CD138-sorted primary samples, using ΔCt, the expression of *miR-340-5p* was plotted against *XIAP*, and a significant inverse correlation was demonstrated

Of the known targets of *miR-340-5p*, overexpression of *miR-340-5p* resulted in significant downregulation of *CCND1* and *NRAS* and all of the predicted target genes, including *MEKK1*, *MEKK2*, *MEKKK3*, *MDM4*, and *XIAP* (Fig. 5a). Moreover, by Western blot, protein expression level of XIAP, which is an inhibitor of apoptosis, and p-ERK1/2, which is a key effector downstream to NRAS, MEKK1, and MEKKK3 in MAPK signaling, were decreased by 27% and 60% respectively upon overexpression of *miR-340-5p* (Fig. 5b). Furthermore, in CD138-sorted primary samples (*n* = 37), a higher expression level of *miR-340-5p* was significantly correlated with a lower level of *XIAP* by qRT-PCR (Fig. 5c, *R*^2^ = 0.2466, *P* = 0.002). These data suggested that *miR-340-5p* might exert its tumor suppressive function by regulating multiple oncogenic target genes involved in MAPK signaling and apoptotic pathways.

### Identification of XIAP as a direct target of *miR-340-5p*

**Fig. 6:**
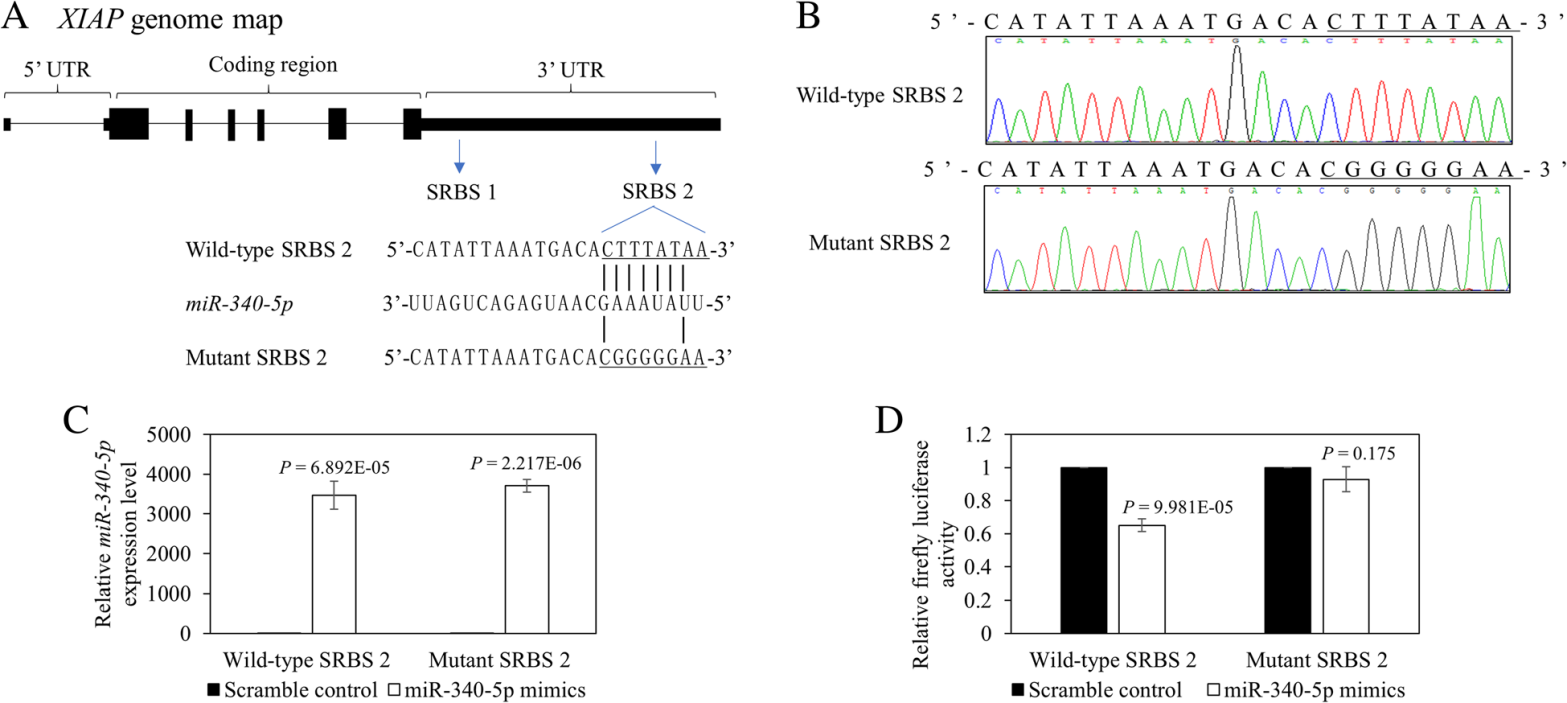
Identification of XIAP as a direct target of *miR-340-5p*. **a** Location, sequence, and predicted binding of *miR-340-5p* seed region to seed region binding site 2 (SRBS 2) on 3′-UTR of *XIAP* mRNA, and the design of mutant sequence. **b** Direct sequencing confirmed the sequences of both wild-type (5′-CTTTATAA-3′) and mutant (5′-CGGGGGAA-3′) in the corresponding luciferase reporter vector. **c** By qRT-PCR, *miR-340-5p* was overexpressed when co-transfection with luciferase reporter vector containing wild-type or mutant SRBS 2. **d** Normalized luciferase activity of luciferase reporter vector carrying wild-type or mutant SRBS 2, in the presence of *miR-340-5p* mimics or scramble control. Error bars represent the standard deviation from three independent transfections

As overexpression of *miR-340-5p* had induced a significant increase of apoptosis in myeloma cells (Fig. 4d), luciferase reporter assay was employed to verify if XIAP, an inhibitor of apoptosis, is a direct target of *miR-340-5p*. By bioinformatics, two conserved *miR-340-5p* SRBSs were identified in the 3′-UTR of *XIAP* (Additional file 2: Figure S2A and Fig. 6a). DNA fragments containing either wild-type or mutant SRBS were generated and cloned into a dual firefly/Renilla luciferase reporter vector (Additional file 2: Figure S2B and Fig. 6b). For each SRBS, luciferase vector containing the wild-type or mutant 3′-UTR of *XIAP* was co-transfected with *miR-340-5p* mimics or scramble control into Hela cells for luciferase assay at 48 h. Upon co-transfection of the wild-type SRBS 1, which was predicted to form 7mer-A1 binding with *miR-340-5p*, overexpression with *miR-340-5p* mimics resulted in comparable luciferase signal as compared with scramble control (Additional file 2: Figure S2C). Moreover, co-transfection of mutant SRBS 1 with *miR-340-5p* mimics had a similar luciferase activity as compared with scramble control (Additional file 2: Figure S2C). In contrast, upon co-transfection with the wild-type SRBS 2, which was predicted to form 8mer binding with *miR-340-5p*, overexpression with *miR-340-5p* mimics (Fig. 6c) led to a reduction of luciferase activity by 34.9%, as compared with scramble control (Fig. 6d; *P* = 9.981E−05). Moreover, upon co-transfection of the mutant SRBS 2 with *miR-340-5p*, the luciferase activity was restored to a comparable level as compared with scramble control (Fig. 6d; *P* = 0.175). Thus, these data suggested that XIAP is a direct target of *miR-340-5p* through binding at SRBS 2 in the 3′-UTR.

## Discussion

There are a number of interesting observations in this study.

Firstly, methylation of *miR-340-5p* in myeloma cell lines and primary myeloma cells was tumor-specific as evidenced by the absence of methylation in normal controls, similar to the tumor-specific methylation of other tumor suppressive miRNAs, such as *miR-124* [34, 35], *miR-203* [24], and *miR-34* family miRNAs [23, 36] in myeloma. This contrasted with the tissue-specific methylation of miRNAs [37, 38], in which miRNA methylation was detected in both tumor cells and the corresponding normal counterparts, and hence likely unimportant in carcinogenesis.

Secondly, in primary samples, methylation of *RNF130/miR-340* appeared as early as MGUS, at a frequency comparable to that of myeloma at diagnosis and relapsed myeloma. Therefore, it is likely that methylation of *RNF130/miR-340* is an early event in the pathogenesis of myeloma, similar to methylation of *miR-203* [24] and *miR-342* [39]. By contrast, *miR-129-2* methylation was implicated in the progression from MGUS to symptomatic myeloma [25], and *miR-34b/c* methylation at relapse or progression of myeloma [23]. Moreover, methylation of *RNF130/miR-340* correlated with shorter OS in newly diagnosed myeloma, similar to *CDKN2A* [40, 41] and *DAPK1* [42] methylation. As this cohort of myeloma patients was uniformly treated [43] with bortezomib-based induction regimen, followed by ASCT, and then thalidomide maintenance, methylation of *RNF130/miR-340* was a potential novel prognostic marker for myeloma and hence warrants a prospective study in larger cohorts of myeloma samples. Similarly, dysregulated expression of a number of miRNAs, such as *miR-135a/b*, *miR-200a/b*, and *miR-596*, has been shown to carry prognostic impact in myeloma [44]; hence, the prognostic significance of *miR-340* expression warrants further investigation.

Thirdly, we showed that the expression of *miR-340-5p* was regulated by promoter hypermethylation of the host gene. First, we showed that *miR-340-5p* expression correlated with that of *RNF130*, consistent with the data showing that intronic miRNAs are transcribed in parallel with their host genes, hence are co-regulated [45]. Similarly, when miRNA expression were correlated with gene expression in myeloma cell lines [46], co-expression of 32 pairs of intronic miRNAs and their host genes were found, including *miR-340/RNF130*. Moreover, we showed low expression of both *miR-340-5p* and *RNF130* in methylated myeloma lines and high expression in unmethylated lines. Furthermore, upon 5-AzadC demethylation, *RNF130* promoter demethylation was associated with re-expression of *miR-340-5p*. Therefore, our data provided additional evidence that intronic miRNAs are silenced by promoter DNA methylation of their host genes [39, 47].

Fourthly, our data demonstrated that *miR-340-5p* is a tumor suppressor in myeloma. This is supported by the downregulation of mRNA of both known targets (*NRAS* and *CCND1*) and bioinformatically predicted targets (*MEKK1*, *MEKK2*, *MEKKK3*, and *XIAP*) upon *miR-340-5p* overexpression. Moreover, by Western blot, the protein level of p-ERK1/2, a key effector of MAPK signaling downstream to NRAS, MEKK1, and MEKKK3, was found repressed upon overexpression of *miR-340-5p*. Therefore, methylation-mediated silencing of *miR-340-5p* may account for the constitutive activation of MAPK signaling in myeloma pathogenesis [48, 49]. Moreover, overexpression of *miR-340-5p* led to downregulation of *CCND1*, which is responsible for the transition from G1 to S phase of the cell cycle. Interestingly, upregulation of D-type cyclins is a unified theme in the pathogenesis of myeloma [50]. As *CCND1* that encodes for cyclin D1 has been proven to be a direct target of *miR-340-5p* by luciferase assay [32], our data of methylation-mediated silencing of *miR-340-5p* may account for the overexpression of CCND1 in myeloma [50]. On the other hand, we showed that XIAP, an inhibitor of apoptosis and an oncogene overexpressed in multiple cancers, is inversely correlated with and a direct target of *miR-340-5p*. These are evidenced by, firstly, the lower expression of *XIAP* correlated with higher expression of *miR-340-5p* in CD138-sorted primary samples and, secondly, by the suppression of luciferase activity upon co-transfection with *miR-340-5p* and the WT *XIAP* 3′ UTR, which was restored by co-transfection with *miR-340-5p* and the mutant *XIAP* 3′ UTR construct. Interestingly, the suppression of XIAP by *miR-340-5p* was mediated by the distal (8mer) but not the proximal (7mer-A1) SRBS, hence consistent with the higher predictive value of SRBS by 8mer than 7mer-A1 [7]. Indeed, high XIAP expression has been demonstrated in primary myeloma plasma cells and cell lines and yielded larger tumors than myeloma cells with XIAP knock-down in a mouse xenograft model [51]. Therefore, the tumor suppressor activity of *miR-340-5p* was mediated by targeting, and hence downregulation, of XIAP in myeloma. Collectively, these results indicated that *miR-340-5p* is a tumor suppressor miRNA in myeloma by inhibition of MAPK signaling, cell proliferation, and induction of apoptosis. Conversely, methylation-mediated silencing of *miR-340-5p* enhanced myeloma plasma cell proliferation via upregulation of CCND1 and MAPK signaling (NRAS/MEKK1/MEKKK3/p-ERK) in addition to enhancing myeloma cell survival by targeting XIAP. Similarly, *miR-340* has been shown to carry tumor suppressor function by inhibiting myeloma cell-induced angiogenesis upon co-culture of myeloma cells with exosomes enriched in *miR-340* [52].

## Conclusions

In conclusion, in myeloma, methylation-mediated silencing of *miR-340-5p* is tumor-specific, reversible, associated with inferior OS, and likely an early event in myelomagenesis. Moreover, *miR-340-5p* potentially exerts its tumor suppressive function via regulation of MAPK signaling and apoptotic pathways.

## Methods

### Patient information

Bone marrow samples were obtained from patients with MGUS (*n* = 18), newly diagnosed myeloma (*n* = 63), and myeloma relapse from complete remission (*n* = 30). Diagnosis of myeloma was based on standard criteria of the International Myeloma Working Group (IMWG) [53]. Complete staging work-up consisted of bone marrow examination, skeletal survey, serum and urine protein electrophoresis, and serum immunoglobulin levels. Of the 63 patients with newly diagnosed myeloma, there were 28 females and 35 males, with a median age of 58 (33–83) years. Apart from 1 patient lacking International Staging System (ISS) data [54], there were 20 stage I, 16 stage II, and 26 stage III cases. There were 8 IgA, 32 IgG, 5 IgD, 14 light chain, and 4 non-secretary myelomas. According to the criteria of the European Group for Blood and Marrow Transplantation Myeloma Registry [55], “relapse” was defined as the reappearance of the same paraprotein detected by serum/urine protein electrophoresis, appearance of new bone lesion or extramedullary plasmacytoma, or unexplained hypercalcemia after prior complete remission. The study has been approved by the Institutional Review Board of Queen Mary Hospital (UW 05-269 T/932), and written informed consent was obtained from patients for the publication of this article and any accompanying data or images.

### Treatment

Transplant-eligible patients with newly diagnosed myeloma were uniformly treated with four cycles of bortezomib-based induction [VTD (bortezomib 1.3 mg/m^2^/dose on days 1, 8, 15, and 22; thalidomide 200 mg/day; and dexamethasone 40 mg/day on days 1, 8, 15, and 22)], followed by autologous stem cell transplant (ASCT) conditioned with melphalan 200 mg/m^2^, and then thalidomide maintenance at a dose of 50 mg/day for 12 months [43]. Treatment response was defined in accordance to the IMWG criteria [56].

### Cell culture

Human myeloma cell lines (HMCLs) KMS-12-PE, MOLP-8, OPM-2, and U-266 were obtained from Deutsche Sammlung von Mikroorganismen und Zellkulturen (DSMZ, Braunschweig, Germany). NCI-H929 was purchased from American Type Culture Collection (ATCC, Manassas, VA, USA). KMS-11/BTZ and OPM-2/BTZ were acquired from Kyowa Hakko Kirin Co. Ltd. (Tokyo, Japan). LP-1 and RPMI-8226 were kindly provided by Prof. Robert Orlowski (Department of Lymphoma/Myeloma, Division of Cancer Medicine, The University of Texas MD Anderson Cancer Center, Houston, TX, USA). JJN-3, OCI-MY5, and RPMI-8226R were kindly provided by Prof. Wee Joo Chng (Department of Medicine, Yong Loo Lin School of Medicine, National University of Singapore). WL-2 was kindly provided by Prof. Andrew Zannettino (Myeloma Research Programme, The University of Adelaide, Australia). MMLAL [57] and MMKKF (unpublished) were established from the myelomatous pleural effusion of myeloma patients. Cell lines were cultured in RPMI-1640 medium (IMDM for LP-1, DMEM + IMDM for MMLAL), supplemented with 10% or 20% fetal bovine serum, 50 U/mL of penicillin, and 50 μg/mL streptomycin, in a humidified atmosphere of 5% CO_2_ at 37 °C. All culture reagents were purchased from Invitrogen (Carlsbad, CA, USA). Cell lines obtained from DSMZ and ATCC have been authenticated using short tandem repeat DNA profiling analysis by them. Cells were used for biology assay within 10 passages from thawing.

### Methylation-specific polymerase chain reaction (MSP)

Genomic DNA was extracted using QIAamp DNA Blood Mini Kit (Qiagen, Hilden, Germany), bisulfite-converted using EpiTect Bisulfite Kit (Qiagen), and hence templates for methylated MSP (M-MSP) and unmethylated MSP (U-MSP). Primer sequences and conditions are in Table 1. Detailed procedures of MSP have been previously described [39, 47].

**Table 1:** Primer sequences and PCR reaction conditions

Primer set	Forward primer (5′–3′)	Reverse primer (5′–3′)	Product size (bp)	MgCl_2_/Tm/cycles	Reference
(I) Methylation-specific PCR (MSP) of *RNF130/miR-340*					
M-MSP	TTT CGT AAA TTT TTC GGG TTT TAC	CCG CTA ATC TAA CGA CAA CG	184	2.0 mM/58 °C/38	–
U-MSP	TTT TGT AAA TTT TTT GGG TTT TAT	CCC CAC TAA TCT AAC AAC AAC A	186	2.0 mM/56 °C/38	–
(II) Quantitative reverse transcription PCR (qRT-PCR)					
*MDM2*	GTC GGA AAG ATG GAG CAA G	TGT GAG GTG GTT ACA GCA	311	60 °C/40	–
*NFKB1*	GGC AGC ACT ACT TCT TGA CC	CAG CAA ACA TGG CAG GCT AT	88	60 °C/40	[58]
*NRAS*	CGC ACT GAC AAT CCA GCT AA	TCG CCT GTC CTC ATG TAT TG	177	60 °C/40	[30]
*GAPDH*	ACC ACA GTC CAT GCC ATC ACT	TCC ACC ACC CTG TTG CTG TA	452	60 °C/40	–
(III) Cloning of luciferase reporter constructs with wild-type SRBSs of *miR-340-5p*					
SRBS 1	AAG GGC TAG CTG TGG TTT CTC TTC GGG GA	AGG GTC GAC GTT TTG CAG GCG TTT GAA CA	214	1.5 mM/60 °C/40	–
SRBS 2	AAG GGC TAG CTG GGA CAT AGT TTG AAG GTG A	AGG GTC GAC TGG ATG CAA AAC CCA CAG AT	213	2.0 mM/56 °C/35	–

*M-MSP* methylated MSP, *U-MSP* unmethylated MSP, *Tm* annealing temperature, *SRBS* seed region binding site

### Quantitative bisulfite pyrosequencing

With bisulfite-treated DNA of HMCLs as a template, specific PCR product overlapping the MSP amplicon was amplified by a pair of methylation-unbiased primers using PyroMark PCR Kit (Qiagen). Primer sequences are as follows: forward primer: 5′-GGG TTT TAG GAG GGT TGT AGA A-3′; reverse primer: 5′-biotin-AAA CAT CCC CCA TCC ATA ATA T-3′; condition: 2 mM MgCl_2_/56 °C/45 cycles. PCR product was purified, and consecutive CpG dinucleotides were pyrosequenced with sequencing primer: 5′-GTG TTG GTT GTT TTT TTG TTA-3′ or 5′-TGT TTT TTT GTT AGG AGT GAT AGT-3′.

### 5-Aza-2′-deoxycytidine (5-AzadC) treatment

WL-2 and RPMI-8226R cells, which were completely methylated for *RNF130/miR-340*, were treated with 0.5 μmol/l or 1 μmol/l 5-AzadC (Sigma-Aldrich, St. Louis, MO, USA) in fresh medium replaced every 24 hours for 6 days. Cells were harvested for DNA and RNA extraction on both days 3 and 6. The relative expression level of *miR-340-5p* in the 5-AzadC-treated group against the untreated group was calculated by the 2^-∆∆CT^ method.

### Quantitative reverse transcription polymerase chain reaction (qRT-PCR)

Total RNA was isolated using the mirVana™ miRNA Isolation Kit (Ambion). For *miR-340-5p*, reverse transcription was performed using the TaqMan MicroRNA RT Kit (ABI, Waltham, MA, USA), followed by qRT-PCR using TaqMan assay (ABI), with *RNU48* as the endogenous control. The expression of *miR-340-5p* were analyzed by the ∆CT method. Correlation between methylation and expression of *miR-340-5p* was calculated by Student’s *t* test. For other genes, reverse transcription was performed using the QuantiTect Reverse Transcription Kit (QIAGEN). *RNF130*, *MEKK1*, *MEKK2*, *MEKKK3*, *XIAP*, *MDM4*, and *CCND1* were quantified using TaqMan assays (ABI). *MDM2*, *NFKB1*, *and NRAS* were quantified using SYBR Green Master Mix (ABI). *GAPDH* was used as the endogenous control. Primers are listed in Table 1. The expression of these genes were calculated by the ∆CT method. Relative expression of gene in response to overexpression of *miR-340-5p* as compared with scramble control was analyzed by the 2^−∆∆CT^ method.

### *miR-340-5p* overexpression

Either *miR-340-5p* mimics or scramble oligonucleotides (negative control) (Ambion, Austin, TX, USA) were transfected into 0.5 × 10^6^/ml myeloma cells (KMS-12-PE, WL-2, and RPMI-8226R) at a final concentration of 75 nM using Lipofectamine 2000 Transfection Reagent (Invitrogen), according to the manufacturers’ instructions.

### Trypan blue exclusion assay

At day 5 after transfection of *miR-340-5p* mimics or scramble control, cell death was analyzed by trypan blue (Sigma-Aldrich, St. Louis, MO, USA). Cells in five random microscopic fields were counted for each sample under a microscope. Dead cell (stained in blue) percentage is equal to the average number of dead cells per microscopic field over the average number of total cells per microscopic field. Data represents the mean dead cell percentage derived from three independent transfections with triplicate in each.

### MTS assay

The number of viable cells in proliferation was measured by CellTiter 96® AQ_ueous_ One Solution Cell Proliferation Assay Kit (Promega, Madison, WI, USA) following the manufacturers’ instructions. The reagent contains a yellowish tetrazolium compound [3-(4,5-dimethylthiazol-2-yl)-5-(3-carboxymethoxyphenyl)-2-(4-sulfophenyl)-2H-tetrazolium, inner salt; MTS], which can be bioreduced by live cells into a purple-colored formazan product that is soluble in tissue culture medium and measurable by the colorimetric method. Relative proliferation percentage of *miR-340-5p* overexpressed cells compared with scramble control was calculated. Data represents the mean relative proliferation percentage derived from three independent transfections with four replicates in each.

### Annexin V/propidium iodide (PI) staining assay

Cell apoptosis was tested by flow cytometry using FITC Annexin V Apoptosis Detection Kit I (BD Biosciences, San Jose, CA, USA). Cells in early apoptosis (suggested by FITC Annexin V positive, PI negative) and late apoptosis (suggested by FITC Annexin V positive, PI positive) are combined as apoptosis cells. Flow cytometry analysis was performed by Cytomics FC 500 Flow Cytometer (Beckman Coulter, Indianapolis, IN, USA).

### Western blot

WL-2 cells transfected with *miR-340-5p* mimics or scramble oligonucleotides control were harvested and lysed in RIPA buffer (Cell Signaling, Danvers, MA, USA). For each sample, cell lysate containing 10 μg protein was separated on Mini-PROTEAN TGX™ 10% SDS-PAGE gel (Bio-Rad, Hercules, CA, USA) and blotted onto a 0.45 μm PVDF membrane (GE Healthcare, Chicago, IL, USA). The membrane was blocked and cut according to protein molecular weight, then incubated in primary antibodies including anti phospho-ERK1/2 (p-ERK1/2; 1:1000; Cell Signaling), anti-total ERK1/2 (t-ERK1/2; 1:1000; Cell Signaling), anti XIAP (1:1000; Cell Signaling), and anti β-actin (1:2000; Cell Signaling) at 4 °C overnight. Thereafter, the membranes were washed and incubated with anti-rabbit horseradish peroxidase conjugate secondary antibody at room temperature for 1 h, followed by imaging on X-ray film. Protein bands were quantified using densitometry as measured by Quantity One 4.6.2 software (Bio-Rad).

### Plasmid constructs

The 3′-UTR of *XIAP* contains two putative SRBSs of *miR-340-5p* [SRBS1: position 863-869 of 3′-UTR (proximal); SRBS2: position 4927-4934 of 3′-UTR (distal)]. For each SRBS, a wild-type DNA segment of ~ 200 bp was amplified and cloned into the NheI and SalI sites of a dual firefly/Renilla luciferase reporter vector, pmirGLO (Promega). Mutant DNA segment of each SRBS was synthesized as gBlocks Gene Fragments purchased from Integrated DNA Technologies (Coralville, IA, USA), followed by PCR amplification and cloned into pmirGLO (Promega). The PCR primers are listed in Table 1. Sequencing was employed to confirm both wild-type and mutant constructs.

### Luciferase assay

In 24-well plate, 0.5 μg luciferase reporter construct with either wild-type or mutant *XIAP* 3′-UTR was co-transfected with 50 nM of either *miR-340-5p* mimics or scramble oligonucleotide control into HeLa cells (a kind gift from Dr. Zou, Department of Medicine, The University of Hong Kong) using Lipofectamine 2000 (Invitrogen). After 48 h, the luminescent signal was generated and analyzed by Dual-Luciferase Reporter Assay System (Promega). Firefly luciferase activity was normalized by Renilla luciferase activity. Relative luciferase activity of *miR-340-5p* overexpressed cells compared with scramble control was calculated. Data represents the mean relative luciferase activity derived from three independent transfections with triplicate in each.

### Statistical analysis

The association of *RNF130/miR-340* methylation with categorical variables including gender, ISS, and immunoglobulin type was studied by chi-square test, and continuous variable, such as age, by Student’s *t* test. Overall survival (OS) was measured from the date of diagnosis to the date of last follow-up or death. OS of patients with and without *RNF130/miR-340* methylation was compared. Survival was plotted by the Kaplan-Meier method and compared by the log-rank test. Cox regression multivariate analysis was performed to analyze the impact of risk factors including *RNF130/miR-340* methylation, age, gender, ISS stage, isotype, and high-risk karyotypes [del(17p), t(4;14), and t(14;16)] on OS using Statistical Package for the Social Sciences (SPSS) version 19.0. The differences between WL-2 cells transfected with *miR-340-5p* mimics and scramble oligonucleotide control in trypan blue exclusion assay and MTS assay were studied by Student’s *t* test. All *P* values were two-sided, and *P* < 0.05 was defined as a significant difference.

## Additional files

Additional file 1: **Figure S1.** Quantitative bisulfite pyrosequencing analysis of *RNF130*/*miR-340* in myeloma cell lines. By detecting the CpG dinucleotides overlapping MSP primer location, the pyrograms of normal, 50% and positive control showed an expected percentage of methylation. Meanwhile, the pyrograms of cell lines showed consistent result with MSP. (TIF 765 kb)

Additional file 2: **Figure S2.** Luciferase assay for SRBS 1. (A) Location and sequence of predicted *miR-340-5p* seed region binding site 1 (SRBS 1) on *XIAP* 3′UTR, and the designed mutant sequence. (B) Direct sequencing result showed *miR-340-5p* SRBS 1 was successfully mutated from 5′-TTTATAA-3′ to 5′-GGGGGAA-3′. (C) pmirGLO luciferase constructs containing wild-type or mutant SRBS 1 were co-transfected with *miR-340-5p* mimics or scramble control into HeLa cells. Luciferase activity was measured at 48 h after transfection. (TIF 610 kb)

Additional file 3: **Figure S3.** Schematic diagram adapted from the UCSC Genome Browser showed the genomic organization of *miR-340* and its host gene *RNF130* on chromosome 5q35. The promoter region, as indicated by the enrichment of H3K4me3 and H3K27ac, was shown embedded in a CpG island (solid green box). (TIF 325 kb)

## Abbreviations

5-AzadC: 5-Aza-2′-deoxycytidine
HMCLs: Human myeloma cell lines
MGUS: Monoclonal gammopathy of undetermined significance
miRNA: MicroRNA
MM: Completely methylated
M-MSP: Methylated MSP
MSP: Methylation-specific PCR
MU: Partially methylated
OS: Overall survival
PCR: Polymerase chain reaction
qRT-PCR: Quantitative real-time PCR
U-MSP: Unmethylated MSP
UU: Completely unmethylated
