# Supplementary figures and images for: Epigenetic silencing of miR-340-5p in multiple myeloma: mechanisms and prognostic impact
Source: Clin Epigenetics. 2019 May 7;11:71. doi: 10.1186/s13148-019-0669-2 (PMC6505104; doi:10.1186/s13148-019-0669-2)

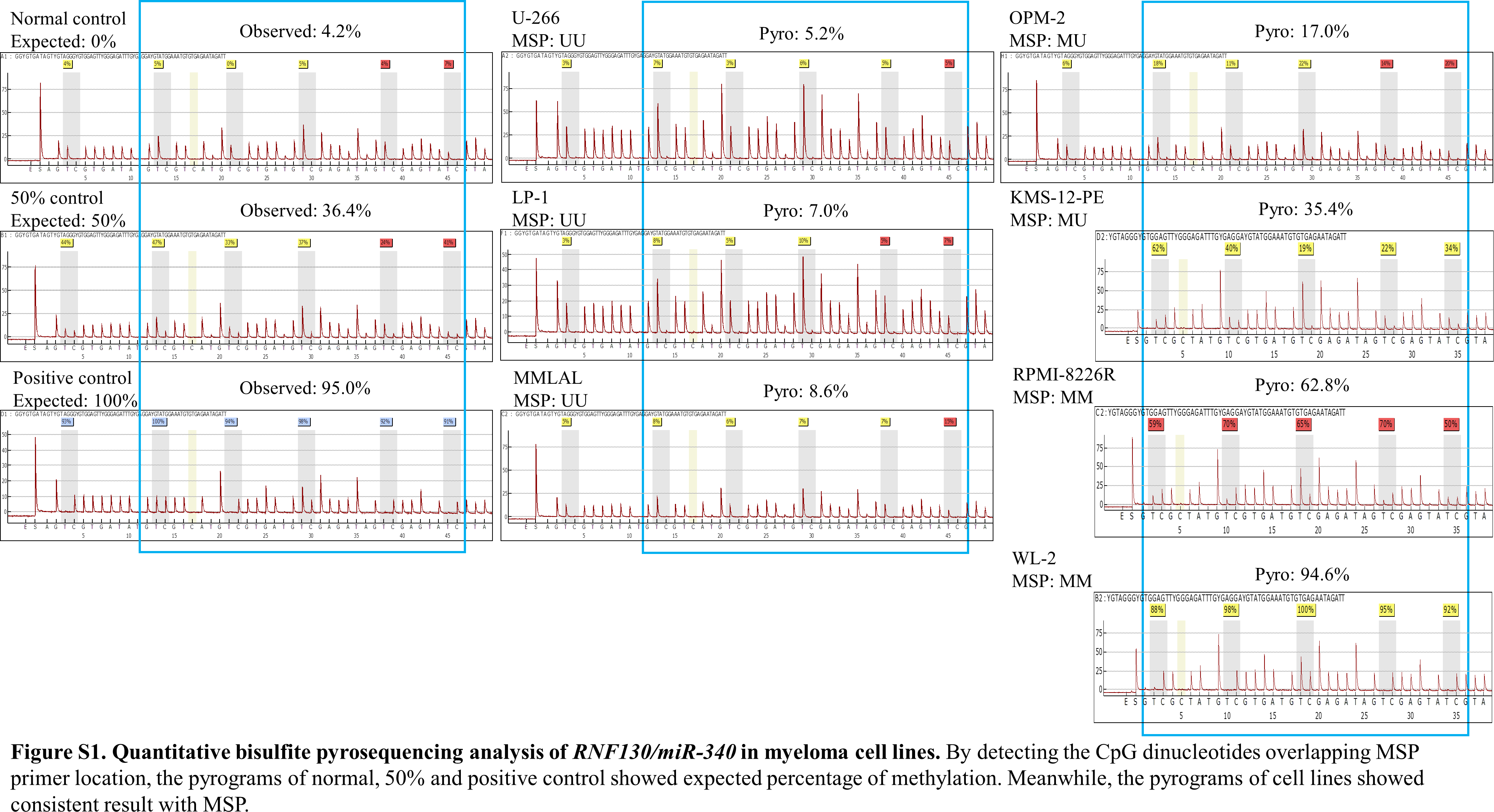

Supplement: Supplementary file 1 — Figure S1. Quantitative bisulfite pyrosequencing analysis of RNF130/miR-340 in myeloma cell lines. By detecting the CpG dinucleotides overlapping MSP primer location, the pyrograms of normal, 50% and positive control showed an expected percentage of methylation. Meanwhile, the pyrograms of cell lines showed consistent result with MSP. (TIF 765 kb) [file 13148_2019_669_MOESM1_ESM.tif]

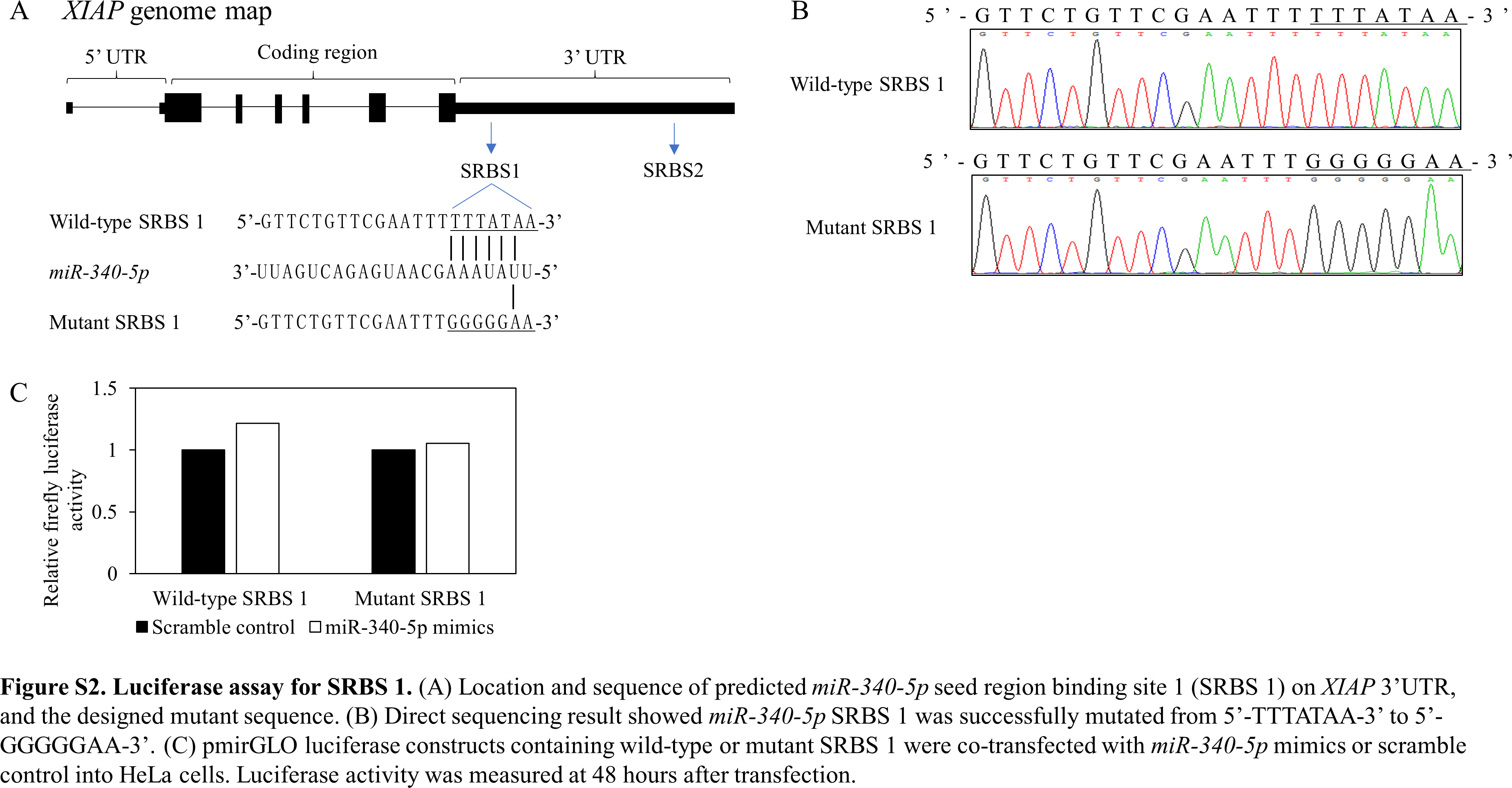

Supplement: Supplementary file 2 — Figure S2. Luciferase assay for SRBS 1. (A) Location and sequence of predicted miR-340-5p seed region binding site 1 (SRBS 1) on XIAP 3′UTR, and the designed mutant sequence. (B) Direct sequencing result showed miR-340-5p SRBS 1 was successfully mutated from 5′-TTTATAA-3′ to 5′-GGGGGAA-3′. (C) pmirGLO luciferase constructs containing wild-type or mutant SRBS 1 were co-transfected with miR-340-5p mimics or scramble control into HeLa cells. Luciferase activity was measured at 48 h after transfection. (TIF 610 kb) [file 13148_2019_669_MOESM2_ESM.tif]

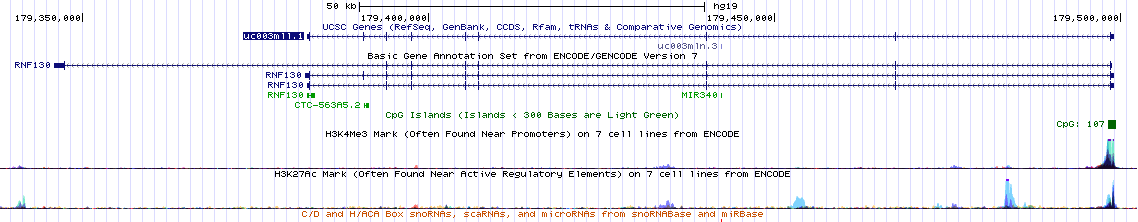

Supplement: Supplementary file 3 — Figure S3. Schematic diagram adapted from the UCSC Genome Browser showed the genomic organization of miR-340 and its host gene RNF130 on chromosome 5q35. The promoter region, as indicated by the enrichment of H3K4me3 and H3K27ac, was shown embedded in a CpG island (solid green box). (TIF 325 kb) [file 13148_2019_669_MOESM3_ESM.tif]
